# Three semi-synthetic approaches to a set of curdlan sulfate polysaccharides with different sulfation patterns

**DOI:** 10.3389/fmolb.2025.1635564

**Published:** 2025-09-10

**Authors:** Fabiana Esposito, Agnieszka Zabłocka, Serena Traboni, Alfonso Iadonisi, Sabina Górska, Emiliano Bedini

**Affiliations:** ^1^ Department of Chemical Sciences, University of Naples Federico II, Complesso Universitario Monte S.Angelo, Napoli, Italy; ^2^ L. Hirszfeld Institute of Immunology and Experimental Therapy, Polish Academy of Sciences, Wrocław, Poland

**Keywords:** glycans, curdlan, semi-synthesis, regioselective sulfation, sulfation pattern, immunological assays

## Abstract

**Introduction:**

Curdlan is a linear homopolysaccharide composed of β-1→3-linked glucose units. It is extracted from some bacteria as an exopolysaccharide and employed in the food industry due to its remarkable rheological and thermal behaviors. Furthermore, its ability to form gel encapsulations with several drugs and its roles in innate and adaptive immunity have fueled an increasing interest in pharmaceutical applications of curdlan and its derivatives. Among them, curdlan sulfate derivatives disclosed not only a highly enhanced water solubility concerning native curdlan but also an efficient immunomodulatory potential in both *in vitro* and *in vivo* assays.

**Methods:**

To detect the effects of the sulfation degree and sulfation pattern on the immunological activity of curdlan sulfate, a set of regioselectively sulfated curdlan polysaccharides was semi-synthesized and fully characterized utilizing nuclear magnetic resonance (NMR) spectroscopy techniques.

**Results:**

Although some regioselectively sulfated curdlan derivatives were already reported some years ago, in this work, a comprehensive semi-synthetic study was developed by investigating three different, complementary approaches based on direct regioselective sulfation or desulfation reactions or multistep protection–sulfation–deprotection procedures.

**Discussion:**

Some of the semi-synthesized curdlan sulfate derivatives were selected as representatives of different sulfation degrees and patterns and subjected to a panel of immunological assays to define some structure–activity relationships.

## 1 Introduction

Curdlan is a biopolymer extracted from some bacteria, with a homopolysaccharide structure composed of β-1→3-linked glucose (Glc) units (McIntosh et al., 2005). It is currently available in high yield from the fermentation of mutant *Agrobacterium* strains (Kalyanasundaram et al., 2012; Zhan et al., 2012). It is mainly used as a food additive due to its remarkable rheological and thermal properties. In addition, a growing interest for the biomedical applications of curdlan (Chaudhari et al., 2021), particularly in the field of immunology, is due to the identification of the effects of β-glucans in modulating both immune and adaptive responses (Goodridge et al., 2009) through the recognition by several receptors, including complement receptor-3 (CR3), Toll-like receptors-2, 4, and 6 (TLR2, TLR4, and TLR6), and dectin-1 (Zhan et al., 2012).

The biomedical properties of native curdlan have fueled increasing efforts in accessing several derivatives thereof with defined structural modifications (Chen and Wang, 2020; Ganie et al., 2022; Zhang and Edgar, 2014). The most investigated derivatizations on curdlan are the insertion of sulfate and carboxymethyl groups, as they can highly increase the water solubility of the native polysaccharide. Moreover, it has been reported that sulfated curdlan derivatives are able to activate dendritic cells mainly through the TLR2 and TLR4 signaling pathways, and this results in a remarkable adjuvant activity in antitumor and hepatitis B virus immunotherapy both *in vitro* and *in vivo* (Jin et al., 2020; Li et al., 2014).

Sulfated curdlan is a representative of the family of the so-called engineered sulfated polysaccharides (ESPs) (Arlov et al., 2021) that are obtained through chemical, enzymatic, or chemo-enzymatic sulfation of native, unsulfated polysaccharides in order to impart novel and augmented biological properties compared to the latter (Caputo et al., 2019; Wang et al., 2018; Zeng et al., 2019). ESPs are typically obtained by random sulfation reactions in which the regioselectivity of the installed sulfate groups within the repeating unit of the polysaccharide cannot be controlled. However, in the recent years, research workers have put intense efforts into developing methods for the regioselective sulfation of polysaccharides (Bedini et al., 2017). The main reason is that ESPs could act in biological events as analogs of glycosaminoglycans (GAGs), which are sulfated polysaccharides widespread in nature and, above all, in mammals, including humans (Perez et al., 2023). GAGs are able to encode a variety of biological information and, therefore, play key roles in a plethora of physiopathological processes, such as immunity, angiogenesis, cancer, and infectious diseases, through their sulfation pattern, that is, the distribution of the sulfate groups along their polysaccharide backbone (Ricard-Blum et al., 2024).

The regioselective installation of sulfate groups on polysaccharides through chemical methods relies upon three main strategies: (i) sulfation of the most reactive hydroxyls through direct reactions under conditions that are mild enough to control the regioselectivity, (ii) desulfation of the most reactive sulfate groups on a previously obtained persulfated polysaccharide derivative, and (iii) multistep methods based on the regioselective installation of suitable protecting groups on some of the hydroxyls of the polysaccharide, followed by sulfation of the unprotected positions and then by cleavage of the previously installed protecting groups (Bedini et al., 2017). Few scattered examples of these different approaches toward the access to regioselectively sulfated curdlan derivatives can be found in literature (Chaidedgumjorn et al., 2002; Gao et al., 1997; Vessella et al., 2021a; Yoshida et al., 1995). In this work, we report a comprehensive investigation of the three strategies as complementary approaches to a set of sulfated curdlan polysaccharide derivatives with different sulfation patterns. The chemical structure of all the obtained products was fully characterized by 1D- and 2D-NMR spectroscopy techniques. Finally, some of them were selected as representatives of different sulfation degrees (DSs) and patterns (respectively intended as the average number of sulfate groups per polysaccharide repeating unit and their distribution among the different sites within the repeating unit) and subjected to a panel of immunological assays in order to define some structure–activity relationships.

## 2 Materials and methods

### 2.1 Materials

Curdlan was purchased from Biosynth. Commercial-grade reagents and solvents for the chemical modification and structural characterization of curdlan were purchased from Avantor and used without further purification. The term “pure water” refers to water purified by a Millipore Milli-Q Gradient system. High-Glc Dulbecco’s modified Eagle’s medium (DMEM) was purchased from Gibco. Fetal bovine serum (FBS) and antibiotics (penicillin/streptomycin mixture) were purchased from Biowest. *N*-(1-Naphthyl)-ethylenediamine was purchased from Serva Feinbiochemica. Sulfanilamide, sodium nitrite, sulfuric acid, and orthophosphoric acid were purchased from Avantor. The trypsin–EDTA solution and phosphate-buffered saline (PBS) (pH 7.4) were prepared in the General Chemistry Laboratory of the Institute of Immunology and Experimental Therapy, PAS. Bacterial lipopolysaccharide (LPS) obtained from *Escherichia coli* (serotype O55:B5) was prepared in the Laboratory of Microbial Immunochemistry and Vaccines of the Institute of Immunology and Experimental Therapy, PAS. A wild-type mouse bone marrow-derived macrophage (BMDM) cell line (NR-945) was obtained from BEI Resources, NIAID, and the National Institutes of Health (NIH). The human TNFα IL-6 and IL-10 DuoSet ELISA kit was purchased from R&D Systems. Tween-20, TLR4 ligand ultrapure LPS-EB, and bovine serum albumin (BSA) were purchased from Sigma-Aldrich. TLR2 and TLR4 HEK-Blue™ cells, the HEK-Blue detection kit, and TLR2 ligand Pam3CSK4 were purchased from InvivoGen. U0126 and SP600125 inhibitors were obtained from Cell Signaling Technology. JSH23 inhibitor and *S-methylisothiourea hemisulfate salt* (S-MIU) were obtained from Sigma-Aldrich.

### 2.2 General methods

Centrifugations were performed with an Eppendorf Centrifuge 5804 R instrument at 4 °C. Dialysis purifications were conducted on Spectra/Por 3.5 kDa cutoff membranes. Freeze-drying was performed with a 5 Pa Lio 5P 4K freeze dryer. Aliquots of curdlan sulfate derivatives were further purified before immunological assays by sequential filtrations on two Sep-Pak C18 cartridges followed by freeze-drying. NMR spectra were recorded on a Bruker Avance-III HD (^1^H: 400 MHz, ^13^C: 100 MHz) or on a Bruker Avance-III (^1^H: 600 MHz, ^13^C: 150 MHz) instrument—the latter equipped with a cryo-probe—in D_2_O [with acetone as the internal standard, ^1^H: (CH_3_)_2_CO at δ 2.22; ^13^C:(*C*H_3_)_2_CO at δ 31.5] or DMSO-*d*_*6*_ (with solvent isotopic impurity as the internal standard, ^1^H: CHD_2_SOCD_3_ at δ 2.49). Data were processed using the data analysis packages integrated with Bruker TopSpin® 4.0.5 software. Gradient-selected COSY experiments were performed using spectral widths of 6,000 Hz in both dimensions using datasets of 2,048 × 300 points. ^1^H, ^13^C-DEPT-HSQC experiments were conducted in the ^1^H-detected mode via single quantum coherence with proton decoupling in the ^13^C domain using datasets of 2,048 × 300 points and typically from 64 to 96 increments.

### 2.3 Conversion of curdlan into LMW-curdlan

A fine suspension of curdlan (271 mg, 1.67 mmol repeating unit) in pure water (27 mL) was treated with trifluoroacetic acid (TFA, 2.2 mL) and then stirred at 60 °C for 3 h. Thereafter, the reaction mixture was neutralized by the addition of a 33% *v*/*v* NaOH solution in pure water. The mixture was dialyzed and then freeze-dried to obtain the low molecular weight polysaccharide (LMW-curdlan, LMW-C) as a white powder (258 mg, 95% mass yield).

### 2.4 Conversion of LMW-curdlan into derivative 1

LMW-C (57.2 mg, 0.350 mmol) was suspended in dry *N*,*N*-dimethylformamide (DMF, 2.1 mL) and then treated with benzoic anhydride (Bz_2_O, 396 mg, 1.75 mmol) and *N*,*N*-diisopropylethylamine (DIPEA, 1.23 mL, 7.06 mmol). After overnight stirring at room temperature (rt), diisopropyl ether (10 mL) was added to form a white precipitate that was collected by centrifugation at 4,600 *g* for 5 min at 4 °C and then dried under vacuum overnight to obtain derivative **1** (88.4 mg, 154% mass yield).

### 2.5 Typical procedure for selective sulfation

LMW-C (62.9 mg, 0.388 mmol) was suspended in dry DMF (2.5 mL; or dry dimethyl sulfoxide, DMSO, 1.2 mL) and then treated with a 1.10 M solution of sulfur trioxide–*N*,*N*-dimethylformamide complex (SO_3_·DMF; or sulfur trioxide–pyridine complex, SO_3_·py) in dry DMF (or dry DMSO, 880 μL, 0.970 mmol). After stirring for 75 min (or 3 h) at rt (or at 50 °C), the mixture was diluted in pure water (15 mL) and neutralized with sodium carbonate (Na_2_CO_3_). Dialysis, freeze-drying, and further purification of the obtained material by filtration on a Sep-Pak C18 cartridge were followed again by freeze-drying, yielding polysaccharide **CS-4** (108.4 mg, 172% mass yield; or **CS-1–3, 57**) as a white amorphous solid.

### 2.6 Typical procedure for desulfation

Persulfated LMW-C polysaccharide (**CS-6**, 26.1 mg, 70.3 μmol) was dissolved in pure water (3.0 mL) and eluted through a short plug of freshly activated Dowex-H^+^ resin with pure water. The eluate was neutralized with pyridine (py) and freeze-dried to produce a residue (26.3 mg) that was dissolved in dry py (3.5 mL) and then treated with *N*-methyl-*N*-(trimethylsilyl)-trifluoroacetamide [MTSTFA, 76 μL, 0.41 mmol; or *N*,*O*-bis(trimethylsilyl)-acetamide, BTSA]. The mixture was stirred at 70 °C–100 °C overnight. The resulting brown mixture was then cooled to rt, diluted with pure water (5 mL), and then dialyzed. The resulting aqueous solution was treated with freshly activated Dowex-H^+^ resin for some minutes. Thereafter, the resin was filtered off, and the resulting solution was neutralized with a 1 M NaOH aqueous solution and then dialyzed and freeze-dried to obtain **CS-8** (12.0 mg, 46% mass yield; or **CS-9**) as a white amorphous solid.

### 2.7 Typical procedure for sulfation of protected, semi-synthetic intermediates and deprotection

Derivative **1** (81.2 mg, 0.310 mmol; or **2** or **4**) was suspended in dry DMF (1.8 mL) and then treated with a 1.15 M solution of SO_3_·py in dry DMF (5.3 mL, 6.1 mmol). After stirring overnight at 50°C, the mixture was cooled to rt, and then, a saturated NaCl solution in acetone (15 mL) was added. The obtained yellowish precipitate was collected by centrifugation at 4,600 g for 5 min at 4°C and then dissolved in pure water (10 mL) and treated with a 33% *v*/*v* NaOH solution in pure water to adjust the pH to 12. The solution was stirred overnight at rt, and then neutralized with a 1 M HCl aqueous solution, dialyzed, and freeze-dried to obtain the derivative **CS-10** (41.9 mg, 52% mass yield; or **CS-11,12**) as a white amorphous solid.

### 2.8 Cell culture

BMDM cells were cultured in DMEM containing GlutaMAX supplemented with 10% FBS and a standard antibiotic mix of penicillin and streptomycin according to the supplier’s guidelines. Culturing was performed in a humidified incubator at 37 °C with 5% CO_2_ and 95% air. When the cells reached approximately 90% confluence, they were detached using a trypsin–EDTA solution, centrifuged at 150 *g* for 10 min, and then resuspended in fresh growth medium. HEK-Blue™ cells, human embryonic kidney cell line HEK293 stably co-transfected with secreted embryonic alkaline phosphatase (SEAP), and the human (h) TLR2 and TLR4 were cultured in DMEM with 10% FBS (v/v) and selective antibiotics as required in the handling procedure (InvivoGen) in a humidified atmosphere (5% CO_2_, 95% air) at 37°C. The cell cultures were renewed with the use of the PBS and without centrifuging when the confluency reached 80% of the bottle.

### 2.9 Assay for cytokine and nitrite/nitrate generation

BMDM cells were seeded in 48-well plates at a density of 1 × 10^6^ cells/mL and maintained in complete medium for 24 h. The next day, the culture medium was refreshed, and the test compounds were applied alone, simultaneously with LPS (1 μg/mL), or after a 5-h pretreatment period prior to LPS (1 μg/mL) exposure. As a positive control, cells were stimulated with LPS from *Escherichia coli* (serotype O55:B5) applied alone, at a concentration of 1 μg/mL, whereas unstimulated cells served as a negative control. As NO is synthesized by inducible NOS, a selective iNOS inhibitor, i.e., S-MIU (10 µM), was used to check the specificity of NO production. Additionally, to determine the impact of ERK 1/2 and JNK kinases and the NF-κB transcription factor activation on the regulation of NO production, BMDM cells were pre-incubated for 1 h with selective kinase inhibitors, namely, U0126 (20 µM) (for ERK1/2), SP600125 (20 µM), and JSH23 (10 µM) (for NF-κB), and then stimulated with particular samples. After 24 h incubation at 37 °C in a humidified atmosphere with 5% CO_2_, culture supernatants were collected and centrifuged at 6,000 *g* for 5 min at rt. The clarified supernatants were then used to assess cytokine levels and NO production.

### 2.10 Nitric oxide determination

Nitric oxide production was assessed by measuring the nitrite levels in the supernatants of macrophage cell cultures after 24 h incubation with the tested compounds using the Griess reagent colorimetric method (Guevara et al., 1998). In brief, 100 μL of each cell culture supernatant was mixed with an appropriate volume of Griess reagent [0.1% *N*-(1-naphthyl)-ethylenediamine and 1% sulfanilamide in 5% ortho-phosphoric acid]. After incubation at rt for 10 min, the absorbance was measured at 550 nm. Nitrite concentrations were quantified by comparison with a standard curve prepared with sodium nitrite (NaNO_2_) in the range 0 μM–50 μM.

### 2.11 ELISA

Cytokines (TNF-α, IL-6, and IL-10) were determined by enzyme-linked immunosorbent assays using human TNF-α DuoSet ELISA according to the manufacturer’s recommended procedure.

### 2.12 Stimulation of TLR2 and TLR4 receptors

The HEK-Blue™ TLR receptors cells (∼140,000 cells/mL) were suspended in HEK-Blue™ detection medium, seeded in 96-well plates, and stimulated with LMW-C and its derivatives at concentrations of 10 and 100 μg/mL. TLR2 ligand Pam3CSK4 (1 μg/mL) and TLR4 ligand ultrapure LPS-EB (1 μg/mL) were used as the positive controls. The activation of receptors was monitored by determining the real-time hydrolytic activity of the SEAP. The colorimetric reaction developed for 10 h–16 h, and the absorbance at 610 nm was measured.

## 3 Results and discussion

### 3.1 Semi-synthesis of a set of regioselectively sulfated curdlan polysaccharides

The molecular weight (M_w_) estimated for native curdlan ranges from medium to rather high (50–1,000 kDa) (Futatsuyama and Ogawa, 1999). As high-M_w_ sulfated polysaccharides can cause adverse effects (Fonseca et al., 2010), a polysaccharide chain shortening was first performed on curdlan under known, partial acid hydrolysis conditions using 1 M TFA at 60 °C for 3 h (Grandpierre et al., 2008; Prieto et al., 2011). After dialytic purification, the obtained low-M_w_ curdlan polysaccharide (LMW-C) was subjected to regioselective sulfation using three different semi-synthetic approaches.

**SCHEME 1:**
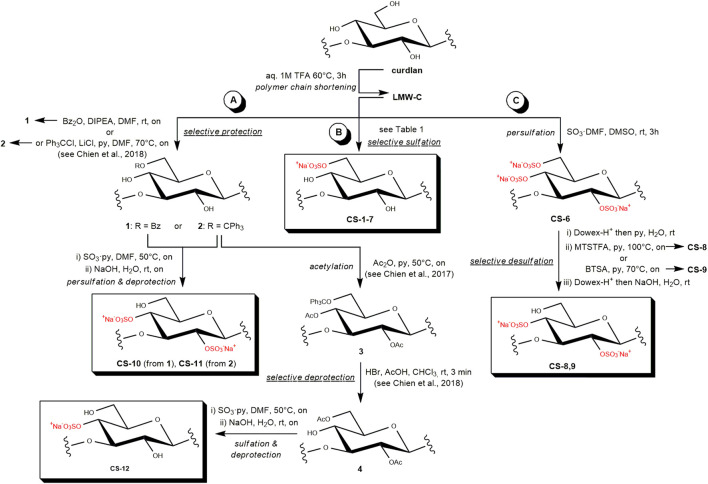
Semi-synthetic strategies toward regioselectively sulfated CS derivatives relying upon **(a)** multistep approaches, **(b)** selective sulfation, and **(c)** selective desulfation reactions.

The first strategy that was investigated in this study was the direct sulfation under mild conditions (Scheme 1). To the best of our knowledge, only a single work previously dealt with the direct, regioselective sulfation of curdlan. It reported the quantitative sulfation of the primary hydroxyls at position C-6 on a LMW-C, together with a partial derivatization on the secondary alcohol at some of the C-2 sites (DS-2 = 0.52–0.55), by employing SO_3_·py as the sulfating agent in dimethyl sulfoxide (DMSO) at rt for significantly short reaction times (60 min–90 min) (Gao et al., 1997). In this work, we tested a sulfation of LMW-C under very similar reaction conditions (Table 1, entry 1) and then screened the effect of three of the main reaction parameters, such as the solvent and sulfating agent type and amount, in order to find the best conditions for regioselective sulfation at Glc O-6 sites with the highest DS (Table 1, entries 3–7). In particular, both SO_3_·py and SO_3_·DMF were tested as sulfating agents in different amounts and in two of the most commonly employed solvents for polysaccharide sulfation reactions, that is, DMSO and DMF (Bedini et al., 2017).

**TABLE 1:** Selective sulfation tests on LMW-C under different reaction conditions (see Scheme 1, pathway B).

Entry	Product	Conditions	Mass yield (%)	Molar yield^a^ (%)	DS-2	DS-4	DS-6	Total DS^b^
1	**CS-1**	SO_3_·py (7 equivalents), DMSO, rt, 75 min	107	74	0	0	0.70^c^ (0.69)^d^	0.70
2	**CS-2**	SO_3_·py (7 equivalents), DMSO, rt, 75 min (x2)	85	59	0	0	0.71^c^	0.71
3	**CS-3**	SO_3_·DMF (7 equivalents), DMSO, rt, 75 min	84	59	0	0	0.68^c^	0.68
4	**CS-4**	SO_3_·DMF (2.5 equivalents), DMF, rt, 75 min	172	74	0.52^e^	0.60^f^	1.00	2.12
5	**CS-5**	SO_3_·py (10 equivalents), DMF, rt, 75 min	87	40	0.30^e^	0.59^f^	1.00	1.89
6	**CS-6**	SO_3_·DMF (10 equivalents), DMF, rt, 3 h	150	52	1.00^e^	1.00^f^	1.00	3.00
7	**CS-7**	SO_3_·py (2.5 equivalents), DMSO, 50 °C, 3 h	108	89	0	0	0.34^c^	0.34

^a^ Calculated from mass yield using the following equation in order to take into consideration the effect of total DS on the average molecular weight (M_w_) of the repeating unit (RU) in the polysaccharide product: $molar\;yield=mass\;yield\times \frac{M_{w}\;\left(curdlan\;RU\right)}{\left.M_{w}\;\left(curdlan\;RU\right)+{\left[total\;DS\times M\right.}_{w}\;\left({SO}_{3}^{-}{Na}^{+}\right)\right]},$ with M_w_ (curdlan RU) = 162 Da and M_w_ (SO_3_^−^Na^+^) = 102 Da.

^b^ Sum of DS-2, DS-4, and DS-6.

^c^ Degree of sulfation at position O-6 of the Glc repeating unit, as calculated by relative integration of signals at 4.36 and 3.94 ppm in the 1H-NMR spectrum (see Figure 1b).

^d^ Degree of sulfation at position O-6 of the Glc repeating unit, as calculated by relative integration of CH_2_-edited signals at δ_H/C_ 4.36,4.24/68.4 and 3.94,3.76/62.0 ppm in the ^1^H, ^13^C-DEPT-HSQC spectrum (see Figure 1a).

^e^ Degree of sulfation at position O-2 of the Glc repeating unit, as calculated by relative integration of the anomeric signals of 2-O-sulfated vs. 2-O-unsulfated Glc residues in the ^1^H, ^13^C-DEPT-HSQC spectrum (see Figure 2; Supplementary Figure S2).

^f^ Degree of sulfation at position O-4 of the Glc repeating unit, as calculated by relative integration of the anomeric signals of 2-O-sulfated vs. 2-O-unsulfated Glc residues in the ^1^H, ^13^C-DEPT-HSQC spectrum (see Figure 2; Supplementary Figure S2).

**FIGURE 1:**
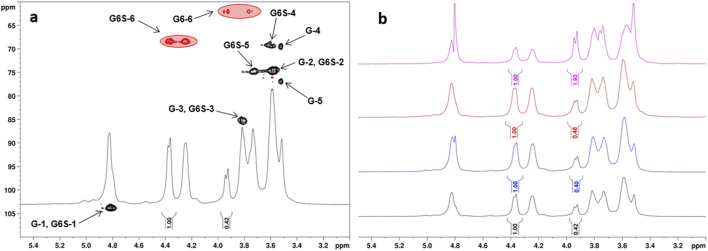
**(a)** ^1^H- and ^1^H, ^13^C-DEPT-HSQC NMR spectra (400 MHz, 298 K, D_2_O) of **CS-1** (G = Glc, G6S = 6-O-sulfated-Glc; DEPT-HSQC signals enclosed in red circles were integrated for DS-6 measurement); **(b)** ^1^H-NMR spectra (400 MHz, 298K, D_2_O) of **CS-1,3** and **CS-7** (from bottom to top).

The obtained set of curdlan sulfate derivatives (**CS-1–7**) was subjected to a detailed structural characterization by means of ^1^H- and 2D-NMR spectroscopy analysis. For product **CS-1**, sulfate groups were detected exclusively at the Glc O-6 sites, as indicated by its ^1^H,^13^C-DEPT-HSQC spectra. Indeed, sulfation is known to induce a 0.7 ppm–0.9 ppm and 6 ppm–11 ppm downfield shift in the ^1^H and ^13^C chemical shift values for hydrogen and carbon atoms, respectively, that are directly linked to the oxygen site carrying the sulfate group (Speciale et al., 2022). In the case of **CS-1**, only CH_2_-edited signals downfield-shifted at ^1^H chemical shift values higher than 4.0 ppm, without any CH-edited signal but the anomeric ones in such a range (Figure 1a). A relative integration of a single diastereotopic methylene signal in the ^1^H-NMR spectrum for both 6-O-sulfated and unsulfated Glc units at 4.36 and 3.94 ppm, respectively, allowed an estimation of the DS-6 value equal to 0.70. A relative integration of the signals associated with methylene signals of 6-O-sulfated and unsulfated Glc units (at δ_H/C_ 4.36, 4.24/68.4 and 3.94,3.76/62.0 ppm, respectively) was also performed on the ^1^H, ^13^C-DEPT-HSQC spectrum of **CS-1**. Notably, the commonly adopted assumption (Esposito et al., 2022; Vessella et al., 2021b) that signals associated with the same CH or CH_2_ atoms in sulfated and unsulfated units display similar ^1^ *J*_C,H_ coupling constants and that a difference of approximately 5 Hz–8 Hz from the experimental set value does not cause a substantial variation of the integrated peak volumes (Gargiulo et al., 2009; Guerrini et al., 2005) was confirmed to work very well in this case, as a DS-6 value equal to 0.69 was measured from the ^1^H, ^13^C-DEPT-HSQC spectrum of **CS-1**. DS-6 measurement was performed for **CS-2, 3** and **CS-7** products too as they displayed ^1^H-NMR spectra very similar to that of **CS-1** (Figure 1b); DS-6 spanned from 0.34 to 0.71. Notably, no significant modification of the DS-6 value measured for **CS-1** was detected either by repeating the reaction twice under such conditions or by changing the sulfating agent (SO_3_·DMF rather than SO_3_·py).

**FIGURE 2:**
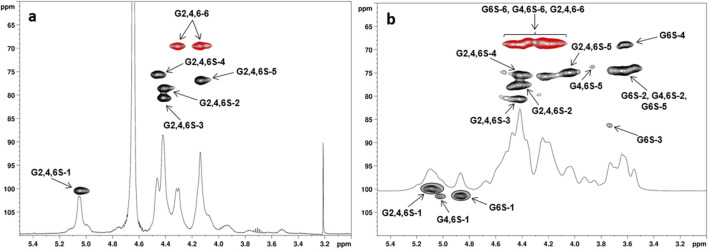
^1^H- and ^1^H, ^13^C-DEPT-HSQC NMR spectra (400 MHz, 298 K, D_2_O) of **(a) CS-6** and **(b) CS-4** with the assignment of the main signals (G6S = 6-O-sulfated-Glc, G4,6S = 4,6-di-O-sulfated-Glc, G2,4,6S = 2,4,6-tri-O-sulfated-Glc; DEPT-HSQC signals enclosed in gray circles were integrated for DS-2 and DS-4 measurement: see also Table 1).

For derivatives **CS-4–6**—obtained with SO_3_·DMF or SO_3_·py in DMF rather than in DMSO—their ^1^H, ^13^C-DEPT-HSQC spectra showed methylene signals only at ^1^H chemical shift values higher than 4.0 ppm, thus demonstrating an exhaustive sulfation of every primary hydroxyl along the polysaccharide backbone. Moreover, the presence of CH-edited signals comprised in a 4.0 ppm–4.6 ppm and 70 ppm–80 ppm range of ^1^H and ^13^C chemical shift values, respectively, clearly suggested the presence of additional sulfate groups derivatizing secondary hydroxyls of the Glc units. In particular, the ^1^H, ^13^C-DEPT-HSQC spectrum of **CS-6** showed only four non-anomeric CH-signals (Figure 2a), all downfield shifted at ^1^H chemical shift values higher than 4.0 ppm (δ_H_ 4.14, 4.41, 4.42, and 4.46 ppm). Together with the presence of a single anomeric signal at δ_H/C_ 5.05/100.4 ppm, it revealed that derivative **CS-6** was per-sulfated, with a quantitative derivatization not only at the O-6 position but also at the O-2 and O-4 sites. Conversely, the ^1^H, ^13^C-DEPT-HSQC spectra of **CS-4** and **CS-5** (Figure 2b; Supplementary Figure S2) showed not only the same signals found for **CS-6** but also some CH-edited signals at ^1^H chemical shift values lower than 4.0 ppm. This suggested a non-quantitative derivatization at the O-2 and O-4 positions of Glc units, which could be confirmed by the presence of multiple signals in the anomeric region. With the help of a ^1^H,^1^H-homonuclear 2D-NMR spectrum such as COSY (Supplementary Figure S3), anomeric signals could be distinguished between the ones related to the 2-O-sulfated and 2-O-unsulfated Glc units. Indeed, all of them but the most ^1^H-downfield shifted one (δ_H_ 5.13 ppm) formed a cross-peak with non-downfield shifted signals related to H-2 atoms of 2-O-unsulfated Glc units. A complete assignment of the signals in the ^1^H, ^13^C-DEPT-HSQC spectrum (Figure 2b), supported by literature data (Vessella et al., 2021a), revealed the presence of tri-, di-, and mono-sulfated Glc units (Glc-2,4,6S, Glc4,6S, and Glc6S). A relative integration of anomeric signals related to 2-O-sulfated vs. 2-O-unsulfated Glc residues afforded an estimation of the DS-2 values indicated in Table 1 (entries 4 and 5). Similarly, DS-4 values were obtained by relative integration of anomeric signals of 4-O-sulfated vs. 4-O-unsulfated Glc units.

**FIGURE 3:**
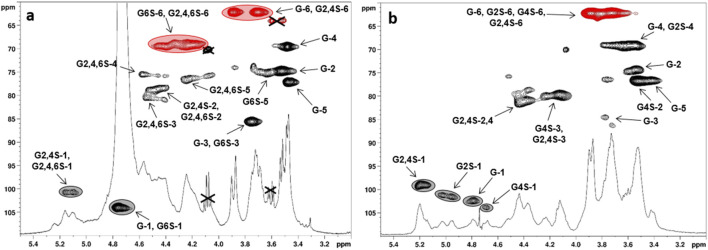
^1^H- and ^1^H, ^13^C-DEPT-HSQC NMR spectra (400 MHz, 298 K, D_2_O) of **(a) CS-8** and **(b) CS-9** with the assignment of the main signals (G = Glc, G2S = 2-O-sulfated-Glc, G4S = 4-O-sulfated-Glc, G6S = 6-O-sulfated-Glc, G2,4S = 2,4-di-O-sulfated-Glc, G2,4,6S = 2,4,6-tri-O-sulfated-Glc; DEPT-HSQC signals enclosed in gray and red circles were integrated for DS-2, DS-4, and DS-6 measurements).

The results discussed above showed that a direct sulfation approach allowed the obtainment, as expected, of some CS derivatives that were regioselectively sulfated at their most reactive sites, that is, primary hydroxyls at the Glc C-6 position. Alternative semi-synthetic strategies had to be conducted to obtain CS products showing a complementary sulfation pattern, that is, with sulfate groups exclusively on secondary hydroxyls of the Glc units. Aiming for this, a regioselective desulfation on persulfated curdlan derivative **CS-6** was tested with BTSA or MTSTFA (Scheme 1c). These are silylating agents already known to selectively cleave sulfate groups at the O-6 position of several polysaccharides and convert the released primary hydroxyl groups into trimethylsilyl ethers. The latter can then be cleaved in turn by an aqueous work-up to restore the alcohol moieties (Bedini et al., 2017). BTSA was already reported for desulfation of a persulfated curdlan polysaccharide; nonetheless, the obtained product was analyzed only by ^1^H-NMR spectrum, which suggested a non-regioselective reaction (Chaidedgumjorn et al., 2002). In order to reinvestigate such reactions and also test MTSTFA as an alternative desulfation agent, **CS-6** was converted into its pyridinium salt by treatment with a strong acid resin followed by neutralization with pyridine, and then subjected to reaction with BTSA or MTSTFA in pyridine at a high temperature. After an aqueous work-up, a pyridinium/Na^+^ cation exchange was performed by treatment with a strong acid resin, followed by neutralization with aqueous NaOH. The obtained products **CS-8, 9** were subjected to detailed structural analysis by ^1^H- and 2D-NMR spectroscopy. The reaction with MTSTFA produced **CS-8**, showing two groups of methylene signals in its ^1^H, ^13^C-DEPT-HSQC NMR spectrum (Figure 3a) at chemical shift values higher or lower than 4.0 ppm, thus corresponding to 6-O-sulfated- and 6-O-desulfated-Glc units, respectively. This demonstrated a non-exhaustive desulfation of the primary positions along the polysaccharide backbone. A relative integration of these signals returned a DS-6 value equal to 0.48 (Table 2, entry 1). Conversely, derivative **CS-9** displayed a single group of methylene signals at δ_H/C_ 3.92, 3.73/62.2 ppm in its ^1^H, ^13^C-DEPT-HSQC NMR spectrum (Figure 3b). This confirmed the complete 6-O-desulfation of **CS-9** (DS-6 equal to 0), whereas sulfate groups at the secondary positions could still be detected, as evidenced by the presence of CH-edited signals at ^1^H chemical shift values higher than 4.0 ppm. In order to gain more insights into the distribution of the residual sulfate groups between the O-2 and O-4 positions of Glc residues, a COSY spectrum (Supplementary Figure S4) was also measured for **CS-9**. With the additional support of literature data (Vessella et al., 2021a), it was possible to assign each of the four anomeric signals detected in the ^1^H, ^13^C-DEPT-HSQC NMR spectrum to Glc units with a different sulfate group pattern. In particular, the two more ^1^H-downfield shifted anomeric signals were assigned to 2-O-sulfated Glc units, with the one at δ_H/C_ 5.20/99.0 ppm being related to 2,4-di-O-sulfated-Glc residues, whereas the other at δ_H/C_ 5.02/101.3 ppm could be assigned to 2-O-sulfated Glc units. Conversely, the two more ^1^H-upfield shifted anomeric signals were assigned to 2-O-unsulfated Glc units. In particular, the most ^1^H-upfield shifted one (δ_H/C_ 4.65/104.0 ppm) could be assigned to 4-O-sulfated Glc residues, whereas the one at δ_H/C_ 4.79/102.4 ppm was related to completely unsulfated Glc units. A relative integration of the four anomeric signals in the ^1^H, ^13^C-DEPT-HSQC NMR spectrum showed an estimation of the relative amounts of Glc units with different sulfation patterns within the **CS-9** polysaccharide backbone. The more abundant units resulted in 2-O-sulfated and 2,4-di-O-sulfated Glc residues (30% and 37%, respectively), followed by Glc (23%) and 4-O-sulfated-Glc (10%) ones. Such relative values suggested a slightly higher reactivity of the sulfate groups at position C-4 rather than that at C-2 toward desulfation with BTSA, and it was summarized by a DS-2 value higher than the DS-4 one (0.67 vs. 0.47, see Table 2, entry 2).

**TABLE 2:** CS products obtained through desulfation or multistep strategies (see Scheme 1, pathways A and C; Vessella et al., 2021a).

Entry	Product	Semi-synthetic strategy	Mass yield (%)	Molar yield (%)	DS-2^a^	DS-4^b^	DS-6^c^	Total DS^d^
1	**CS-8**	Desulfation	46^e^	76^f^	0.34	0.37	0.48	1.19
2	**CS-9**	Desulfation	59^e^	99^f^	0.67	0.47	0	1.14
3	**CS-10**	Multistep	88^g^	55^h^	0.54	0.42	0	0.96
4	**CS-11**	Multistep	40^g^	19^h^	0.20	0.62	1.00	1.82
5	**CS-12**	Multistep	89^g^	37^h^	0.42	1.00	0.81	2.23
6^i^	**CS-13**	Multistep	40	21^h^	0	0.78	0.66	1.44
7^i^	**CS-14**	Multistep	69	38^h^	0	0.76	0.61	1.33
8^i^	**CS-15**	Multistep	48	32^h^	0	0.35	0.46	0.81
9^i^	**CS-16**	Multistep	50	28^h^	0	0.53	0.69	1.22
10^i^	**CS-17**	Multistep	46	27^h^	0.46	0.12	0.53	1.11
11^i^	**CS-18**	Multistep	37	26^h^	0.49	0	0.16	0.65

^a^ Degree of sulfation at position O-2 of the Glc repeating unit, as calculated by relative integration of the anomeric signals of 2-O-sulfated vs. 2-O-unsulfated Glc residues in the ^1^H, ^13^C-DEPT-HSQC spectrum (see Figures 3a,b; Supplementary Figures S5, S7, S8).

^b^ Degree of sulfation at position O-4 of the Glc repeating unit, as calculated by relative integration of the anomeric signals of 2-O-sulfated vs. 2-O-unsulfated Glc residues in the ^1^H, ^13^C-DEPT-HSQC spectrum (see Figures 3a,b; Supplementary Figures S5, S7, S8).

^c^ Degree of sulfation at position O-6 of the Glc repeating unit, as calculated by relative integration of CH_2_-edited signals at δ_H/C_ 4.36, 4.24/68.4 and 3.94, 3.76/62.0 ppm in the ^1^H, ^13^C-DEPT-HSQC spectrum (see Figure 3A; Supplementary Figures S5, S7, S8).

^d^ Sum of DS-2, DS-4, and DS-6.

^e^ Calculated from the **CS-6** starting amount.

^f^ Calculated from mass yield using the following equation in order to take into consideration the effect of total DS on the average molecular weight (M_w_) of the repeating unit (RU) in the polysaccharide product: $molar\;yield=mass\;yield\times \frac{M_{w}\;\left(CS6\;RU\right)}{\left.M_{w}\;\left(curdlan\;RU\right)+{\left[total\;DS\times M\right.}_{w}\;\left({SO}_{3}^{-}{Na}^{+}\right)\right]},$ with M_w_ (**CS-6**) = 468 Da, M_w_ (curdlan RU) = 162 Da and M_w_ (SO_3_^−^Na^+^) = 102 Da.

^g^ Calculated from the LMW-C starting amount.

^h^ Calculated from mass yield using the following equation in order to take into consideration the effect of total DS on the average molecular weight (M_w_) of the repeating unit (RU) in the polysaccharide product: $molar\;yield=mass\;yield\times \frac{M_{w}\;\left(curdlan\;RU\right)}{\left.M_{w}\;\left(curdlan\;RU\right)+{\left[total\;DS\times M\right.}_{w}\;\left({SO}_{3}^{-}{Na}^{+}\right)\right]},$ with M_w_ (curdlan RU) = 162 Da and M_w_ (SO_3_^−^Na^+^) = 102 Da.

^i^ See Vessella et al., 2021a.

By adopting the same structural characterization approach, an estimation of DS-2 and DS-4 could be conducted for **CS-8** too, revealing very similar values (0.34 and 0.37, respectively), which were also rather close to that of DS-6 (0.48, see Table 2, entry 1). This suggested that sulfate groups of 2,4,6-trisulfated-Glc units in persulfated curdlan **CS-6** were cleaved randomly by MTSTFA. Nonetheless, the ^1^H, ^13^C-DEPT-HSQC spectrum of **CS-8** revealed that the polysaccharide backbone was not constituted by all possible Glc sulfation patterns, as indicated by the presence of only two groups of anomeric signals (Figure 3a). Each of them was associated with two distinct Glc residues, that is, 2,4,6-tri-O-sulfated-Glc and 2,4-di-O-sulfated-Glc residues for more ^1^H-downfield shifted signals at δ_H/C_ 5.16/100.6 and 5.10/100.5 ppm and 6-O-sulfated-Glc and completely unsulfated Glc units for more ^1^H-upfield shifted signals at δ_H/C_ 4.74/103.7 and 4.77/103.4 ppm. This suggested that after the first sulfate group is randomly cleaved from a 2,4,6-tri-O-sulfated Glc unit, the reaction proceeds with some preferences, which are as follows: (i) partially desulfated Glc residues are subjected to further desulfation more easily than the persulfated units because the latter can still be detected in the final product despite the major presence of completely unsulfated Glc units, and (ii) Glc units that were already desulfated at a secondary position are more prone to lose the second sulfate group at a secondary site again, as suggested by the absence of 2,6- and 4,6-disulfated-Glc units and the 2- and 4-sulfated ones, whereas both 2,4-disulfated and 6-sulfated residues can be detected as constituents of the **CS-8** backbone.

The third semi-synthetic strategy employed in this work relied upon protection–sulfation–deprotection, multistep approaches. One of the selected protecting groups for the first step was triphenylmethyl (trityl, Tr) ether. Its marked steric hindrance could be exploited to protect only the primary alcohol moiety of Glc units, as the latter are known to be more reactive and less sterically hindered than secondary hydroxyls at C-2 and C-4 in curdlan. Tritylation was conducted under homogeneous conditions already reported for curdlan, utilizing trityl chloride and pyridine in a DMF/LiCl system at 70 °C to obtain known derivative **2** (Scheme 1a) (Chien and Iwata, 2018). An alternative protecting group that was installed on LMW-C was benzoyl ester (Bz). As it is known to protect not only primary but also secondary hydroxyl moieties, a slight adaptation of an organocatalyzed, site-selective method employed for monosaccharides was tested (Ren et al., 2018). This relied upon the use of Bz_2_O as the acylating agent in the presence of DIPEA as the organocatalyst. The obtained derivative **1** was analyzed by ^1^H-NMR spectroscopy in DMSO-*d*_*6*_, which confirmed the presence of signals in the typical region (8.2-7.0 ppm) for aromatic H atoms of benzoyl esters. Notably, the presence of Bz protecting groups in **1** was further confirmed by the detection of aromatic signals in a diffusion-ordered (1D-DOSY) NMR spectrum (Supplementary Figure S1). Indeed, this allows distinguishing between peaks associated to slowly diffusing macromolecules and signals related to low-molecular-weight contaminants—typically faster self-diffusing in solution—as only the former are retained in the 1D-DOSY spectrum, whereas the latter are cut off (Bedini et al., 2023).

Both synthetic intermediates **1** and **2** were subjected to sulfation with SO_3_·py in DMF at 50 °C, followed by deprotection under aqueous alkaline or acid conditions, respectively, to obtain **CS-10** and **CS-11** derivatives. Moreover, tritylated polysaccharide **2** was also subjected to further protection by acetylation of the secondary hydroxyls with acetic anhydride (Ac_2_O) in pyridine at 50 °C to obtain known derivative **3**. The latter was subjected in turn to trityl cleavage with HBr in a chloroform–acetic acid mixture. Under these conditions, a concomitant acetyl migration from the C-4 to C-6 site is known to take place (Chien et al., 2017). Finally, the obtained synthetic intermediate **4** was subjected to sulfation and then acetyl cleavage to obtain product **CS-12**.

The three semi-synthetic derivatives **CS-10–12** resulting from multistep sequences were analyzed by ^1^H- and 2D-NMR spectroscopy in order to confirm or revise their expected structures (Scheme 1a). The ^1^H, ^13^C-DEPT-HSQC of **CS-10** clearly showed the absence of sulfate groups on any of the primary positions along the polysaccharide backbone, as CH_2_-edited signals appeared exclusively in a region of the spectrum at δ_H_ lower than 4.0 ppm (Supplementary Figure S5). The presence of several CH-edited signals, downfield shifted at ^1^H chemical shift values between 4.3 and 4.6 ppm, suggested that sulfate groups were present instead on the secondary sites of Glc units. This result was in agreement with the putative structure of **CS-10**, thus demonstrating that the multistep approach relying upon the Bz protecting groups was successful. Moreover, the assignment of the main signals in the ^1^H, ^13^C-DEPT-HSQC spectrum was possible with the help of a COSY spectrum (Supplementary Figure S6) and literature data (Vessella et al., 2021a). In analogy with the case of products **CS-4, 5** and **CS-8, 9** discussed above, DS-2 and DS-4 values (0.54 and 0.42, respectively: see Table 2, entry 3) could be estimated from the relative integration of the distinct anomeric signals at δ_H/C_ 5.08/100.9, 4.99/101.7, and 4.79/102.4 ppm, which were assigned to 2,4-di-O-sulfated-, 2-O-sulfated-, and unsulfated Glc-CH-1 atoms, respectively. A similar analytical approach was employed for structural characterization of **CS-11** and **CS-12** (Supplementary Figures S7, S8). It resulted in a non-regioselective distribution of sulfate groups among the C-2, C-4, and C-6 sites of Glc units (Table 2, entries 4–5), thus assessing that the multistep strategies relying upon the Tr protecting groups were unsuccessful.

### 3.2 Immunological assays on a set of sulfated curdlan polysaccharides

**FIGURE 4:**
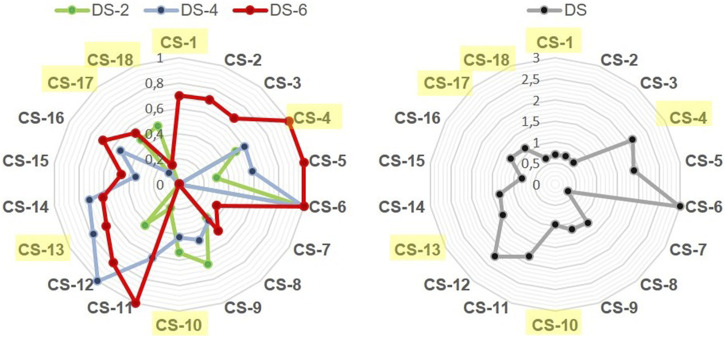
Radar charts show comparisons among compounds CS-1 to CS-18 across four different criteria (DS-2, DS-4, DS-6 and total DS). Compounds CS-1, CS-4, CS-10, CS-13, CS-17, and CS-18 are highlighted in yellow.

The eighteen CS derivatives listed in Tables 1, 2, obtained in this work and our previously published report (Vessella et al., 2021a), represent a significantly comprehensive set of curdlan polysaccharides differentiated for the total DS and the distribution of sulfate groups among the three alcohol positions of Glc units. The use of radar charts, graphically displaying the variation of DS-2, DS-4, DS-6, and total DS (Figure 4), allowed us to select six derivatives (**CS-1, 4, 10, 13, 17,** and **18**) as representatives of different sulfation degrees and patterns. Their structural diversity is crucial as the distribution of the sulfate groups within the Glc repeating unit could significantly influence their biological activity. The selected CS derivatives were, therefore, subjected to a preliminary screening of their immunomodulatory activities in comparison with the starting, unsulfated LMW-C polysaccharide.

As the innate immune response is one of the most important components of the body’s defenses, our study focused on how macrophages respond to curdlan derivatives. As tissue-resident phagocytes, macrophages play a pivotal role in both innate and adaptive immunity. They are the first line of defense against pathogenic microorganisms, including bacteria and viruses. We conducted experiments using a BMDM cell line isolated from wild-type mice. This model was chosen due to the critical yet still not fully studied role of macrophage responses to unsulfated LMW-C polysaccharides and their derivatives.

A key feature of macrophage activation is the production of NO and cytokines, which are crucial factors of the innate immune response. Cytokines such as TNF-α, IL-6, and IL-10 orchestrate the inflammatory response by modulating the signaling, as well as the recruitment and activation of immune cells (Arango Duque and Descoteaux, 2014; Chen et al., 2018). Meanwhile, NO, which is produced via inducible nitric oxide synthase (iNOS), exhibits potent antimicrobial and immunomodulatory properties (Farahani et al., 2025). It plays a dual role in directly eliminating pathogens and regulating inflammation.

**FIGURE 5:**
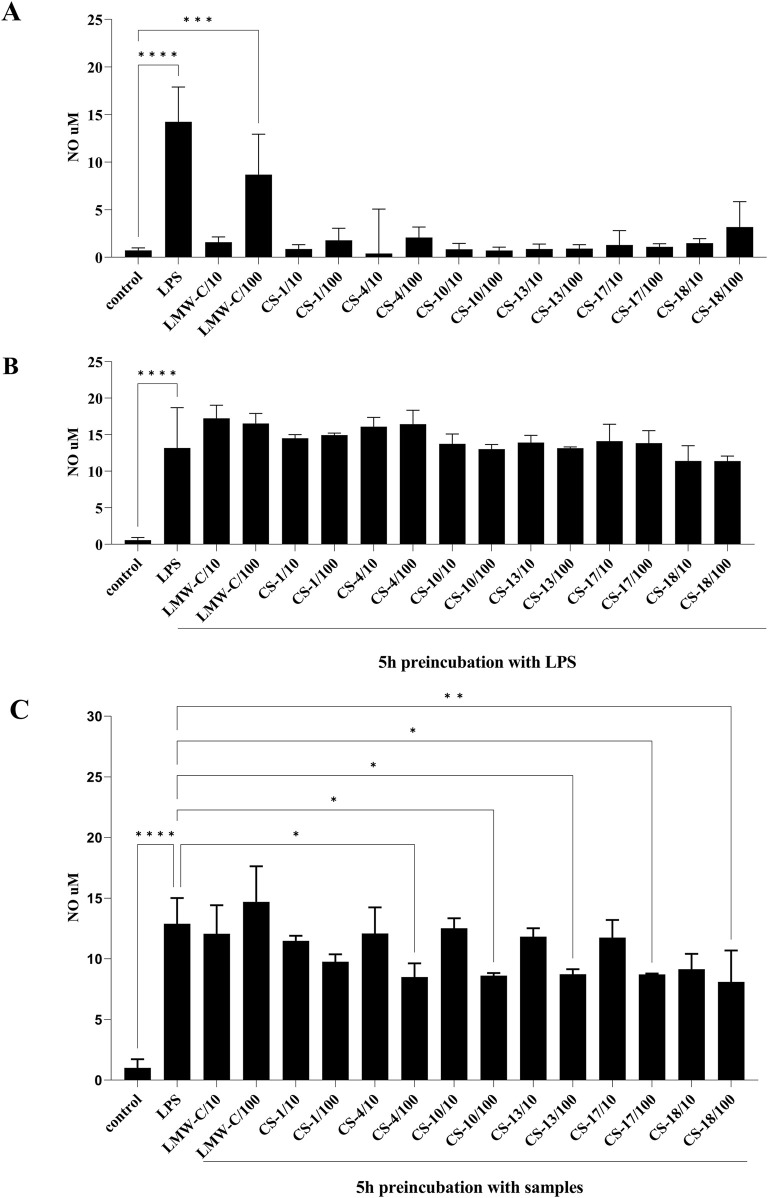
Effect of LMW-C and derivatives **CS-1, 4, 10, 13, 17,** and **18** on nitric oxide production in BMDM cells **(A)**, during inflammation **(B)**, or in a protective model **(C)**. BMDM cells (1 × 10^6^/mL) were **(A)** treated with LMW-C or its derivatives at concentrations of 10 and 100 μg/mL or with LPS (1 μg/mL) as a positive control for 24 h; **(B)** pre-incubated for 5 h with LPS, and then LMW-C or its derivatives at concentrations of 10 and 100 μg/mL were added; **(C)** pretreated with LMW-C or its derivatives at 10 and 100 μg/mL concentrations for 5 h and then stimulated with LPS (1 μg/mL) to induce a pro-inflammatory response. After 24 h, cell culture supernatants were collected, and nitric oxide production was assessed by measuring nitrite levels by the Griess reaction. Statistical analysis was performed by one-way ANOVA. Significant differences in NO levels compared to the untreated control **(A, B)** or LPS **(B, C)** were indicated (*p ≤ 0.05, **p ≤ 0.001, ***p ≤ 0.0005, and ****p ≤ 0.0001). Data represent the mean ± SD of three independent experiments (n = 3).

**FIGURE 6:**
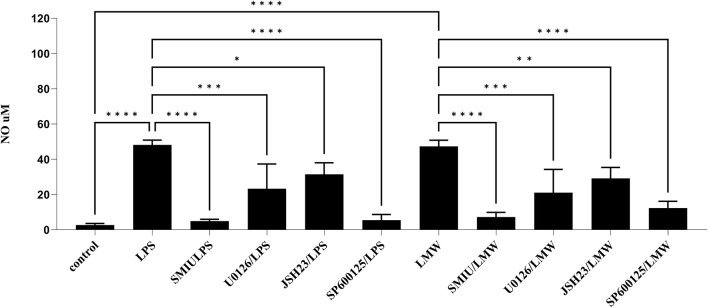
Impact of MAPK/NF-kB/iNOS inhibitors on NO production in BMDM macrophages. To check the specificity of NO production, BMDM cells (1 × 10^6^/mL) were first pretreated for 1 h with selective iNOS inhibitor S-MIU (10 µM), ERK 1/2 inhibitor U0126 (20 µM), JNK inhibitor SP600125 (10 µM), or JSH23 inhibitor (10 µM) and then cultured with LMW-C (100 μg/mL) or the positive control LPS (1 μg/mL) for 24 h in 5% CO_2_/95% air. Thereafter, supernatants were collected, and the level of NO was detected by the Griess reaction. The results represent at least three independent experiments (n = 3–4), and data are presented as the mean ± SD. One-way ANOVA test was used to examine the differences between LPS or LMW-C-treated cells in the presence or absence of inhibitors (*p ≤ 0.05, **p ≤ 0.001, ***p ≤ 0, and ****p ≤ 0.0001).

In our studies, the NO level, as measured by nitrite concentration, was determined using the Griess reaction in BMDM cell culture supernatants following treatment with LMW-C and its derivatives **CS-1, 4, 10, 13, 17,** and **18**. The results showed that LMW-C at a dose of 100 μg/mL (but not at 10 μg/mL) had a clear immunomodulatory effect, significantly increasing NO production by BMDM cells to 8.68 µM compared to that produced by the control cells. However, none of the tested sulfated derivatives exhibited a similar effect (Figure 5A). Pretreating the BMDM cells with the selective iNOS inhibitor S-MIU significantly inhibited NO production, confirming that the observed effect is under the control of iNOS (Figure 6). It is well known that the transcriptional regulation of the *iNOS* gene is mainly regulated at the transcriptional level due to the activation of mitogen-activated protein kinases (MAPKs) and several transcription factors. Among these, NF-κB activation appears to be the most critical event (Arthur and Ley, 2013; Dorrington and Fraser, 2019; Sharif et al., 2007). Therefore, these factors were investigated to better understand LMW-C signaling in macrophages and the potential roles of extracellular signal-related kinase 1/2 (ERK 1/2) and c-Jun N-terminal kinase (JNK, the members of the MAPK family), and the NF-κB transcription factor in LMW-C-induced iNOS expression. Using selective inhibitors of JNK (SP600125), ERK1/2 (U0126, which blocks the kinases MEK-1 and MEK-2 that are crucial for the Ras/Raf/MEK/ERK signaling pathway), and NF-κB (JSH23, which blocks the nuclear translocation of the p65 subunit of NF-κB), a significant reduction in NO production in response to LMW-C was observed. The observed effect for LMW was similar to that of the pathway activated by the classic activator, LPS (Figure 6). These findings demonstrate that LMW-C induces iNOS expression in mouse macrophages through positive signaling mediated by the JNK and ERK1/2 MAPK pathways. Additionally, they confirm the involvement of the NF-κB transcription factor in activating the *iNOS* gene.

To evaluate the potential anti-inflammatory properties of LMW-C and its derivatives, the compounds were administered to the cells 5 h after lipopolysaccharide (LPS, a strong pro-inflammatory agent) application. The analysis of their ability to suppress LPS-induced inflammation revealed no inhibitory effect for either LMW-C or any of its derivatives (Figure 5B). However, pre-incubation of BMDM cells with the investigated LMW-curdlan derivatives for 5 h before LPS stimulation showed a significant and dose-dependent inhibitory effect. **CS-4, CS-10, CS-13, CS-17,** and **CS-18** derivatives, but not **CS-1**, appeared to be effective in modulating LPS-induced NO production compared to native LMW-curdlan, highlighting their enhanced immunomodulatory potential (Figure 5C). Interestingly, **CS-1** has one of the lowest total DSs (0.70) among the tested sulfated derivatives (see Tables 1, 2; Figure 4); nonetheless, the protective effect against the LPS-induced pro-inflammatory response appears to depend not only on the total amount of sulfate groups on the polysaccharide, as derivative **CS-18** shows a dose-dependent effect in spite of a slightly lower total DS value (0.65). The marked difference in sulfation pattern—**CS-1** carries sulfate groups only at the O-6 positions, whereas **CS-18** carries them almost exclusively at the O-2 sites—suggests a role of such a structural feature, as is typically the case for the biological activities of GAGs.

**FIGURE 7:**
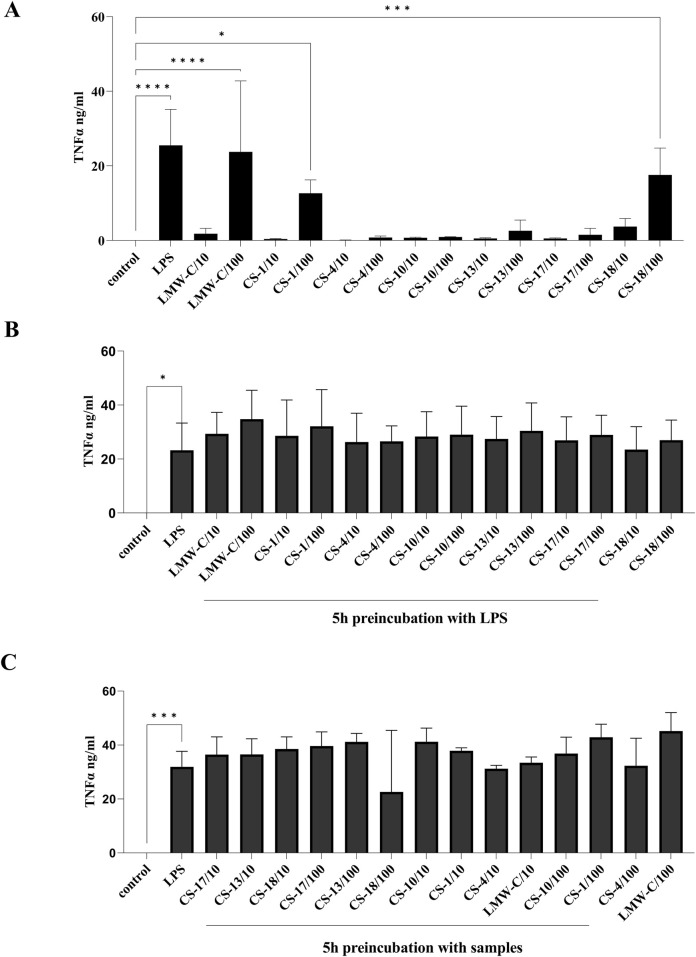
Effect of LMW-C and its derivatives **CS-1, 4, 10, 13, 17,** and **18** on TNF-α production in BMDM cells **(A)**, during inflammation **(B)**, or in a protective model **(C)**. BMDM cells (1 × 10^6^/mL) were **(A)** treated with LMW-C or its derivatives at concentrations of 10 and 100 μg/mL or with LPS (1 μg/mL) as a positive control for 24 h; **(B)** pre-incubated for 5 h with LPS, and then LMW-C or its derivatives at concentrations of 10 and 100 μg/mL were added. LPS alone (1 μg/mL) was used as a reference control; **(C)** pretreated with LMW-C or its derivatives at 10 and 100 μg/mL concentrations for 5 h, and then the cells were stimulated with LPS (1 μg/mL) to induce a pro-inflammatory response. After 24 h, cell culture supernatants were collected, and TNF-α level was measured by ELISA. Statistical analysis was performed by one-way ANOVA. Significant differences in TNF-α levels compared to the untreated control **(A, B, C)** or LPS **(B, C)** were indicated (*p ≤ 0.05, ***p ≤ 0.0005, and ****p ≤ 0.0001). Data represent the mean ± SD of three independent experiments (n = 3–4).

**FIGURE 8:**
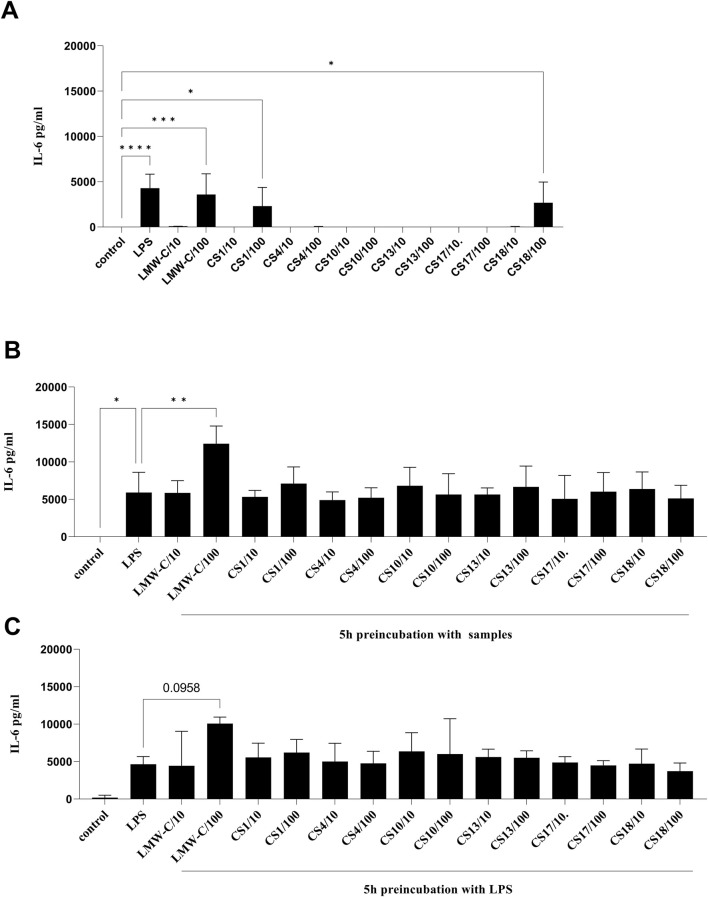
Effect of LMW-C and its derivatives **CS-1, 4, 10, 13, 17,** and **18** on IL-6 production in BMDM cells **(A)**, during inflammation **(B)**, or in a protective model **(C)**. BMDM cells (1 × 10^6^/mL) were **(A)** treated with LMW-C or its derivatives at concentrations of 10 and 100 μg/mL or with LPS (1 μg/mL) as a positive control for 24 h; **(B)** pre-incubated for 5 h with LPS, and then LMW-C or its derivatives at concentrations of 10 and 100 μg/mL were added. LPS alone (1 μg/mL) was used as a reference control; **(C)** pretreated with LMW-C or its derivatives at 10 and 100 μg/mL concentrations for 5 h, and then the cells were stimulated with LPS (1 μg/mL) to induce a pro-inflammatory response. After 24 h, cell culture supernatants were collected, and IL-6 level was measured by ELISA. Statistical analysis was performed by one-way ANOVA. Significant differences in TNF-α levels compared to the untreated control **(A, B, C)** or LPS **(B, C)** were indicated (*p ≤ 0.05, **p ≤ 0.001, ***p ≤ 0.0005, and ****p ≤ 0.0001). Data represent the mean ± SD of at least three independent experiments (n = 3–7).

The immunomodulatory potential of LMW-C and its derivatives were also determined by assessing their effects on the production of the pro-inflammatory cytokine TNF-α, pleiotropic cytokine IL-6, and the anti-inflammatory cytokine IL-10 in BMDM cells. Treatment with LMW-C or its derivatives at a dose of 100 μg/mL increased pro-inflammatory TNF-α (Figure 7A) and pleiotropic IL-6 (Figure 8A) levels. However, this effect was only statistically significant for LMW-C and **CS-1, 18**. There was no effect on anti-inflammatory IL-10 production (data not presented). Furthermore, pre-incubation of BMDM cells with LPS for 5 hours, followed by treatment with samples (Figures 7B, 8B), or pre-incubation of BMDM cells with the tested compounds for 5 hours, followed by treatment with LPS (Figures 7C, 8C), showed that neither LMW-C nor its derivatives inhibited LPS-induced TNF-α and IL-6 production. Conversely, 5 hours of pre-incubation with LMW-C, followed by treatment with LPS, increased the IL-6 levels (p ≤ 0.0958), suggesting its potential modulatory ability in inflammation.

**FIGURE 9:**
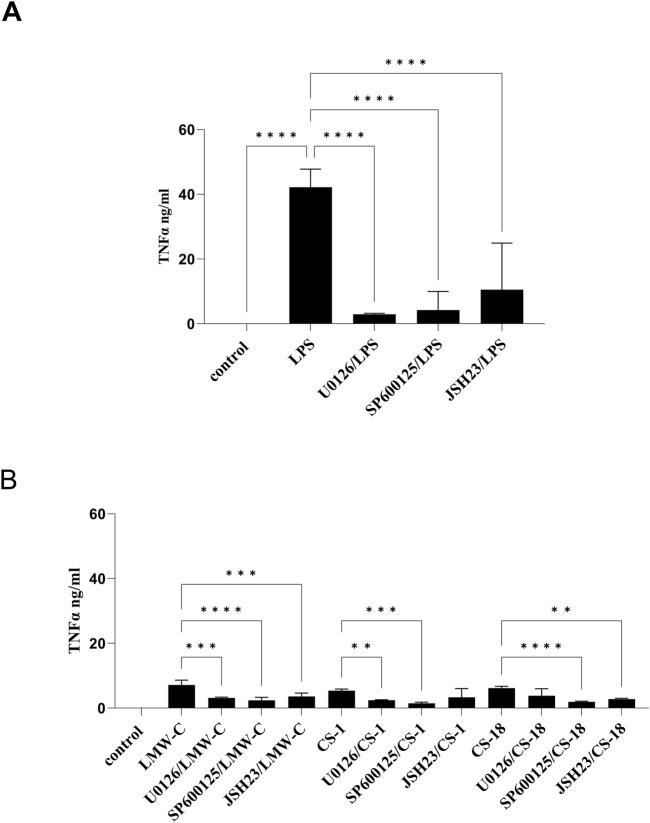
Impact of MAPK/NF-κB/iNOS inhibitors on TNF-α production in BMDM macrophages. To check the specificity of TNF-α production, BMDM cells (1 × 10^6^/mL) were first pretreated for 1 h with selective iNOS inhibitor S-MIU (10 µM), ERK 1/2 inhibitor U0126 (20 µM), JNK inhibitor SP600125 (10 µM), or JSH23 inhibitor (10 µM) and then cultured with positive control LPS (1 μg/mL) **(A)** or LMW-C, **CS-1,** and **CS-18** (100 μg/mL) **(B)** for 24 h in 5% CO_2_/95% air. Thereafter, supernatants were collected, and the level of TNF-α was detected by ELISA. The results represent at least two independent experiments (n = 2–4), and data are presented as the mean ± SD. One-way ANOVA test was used to examine the differences between LPS or LMW-C-treated cells in the presence or absence of inhibitors (**p ≤ 0.001, ***p ≤ 0.001, and ****p ≤ 0.0001).

**FIGURE 10:**
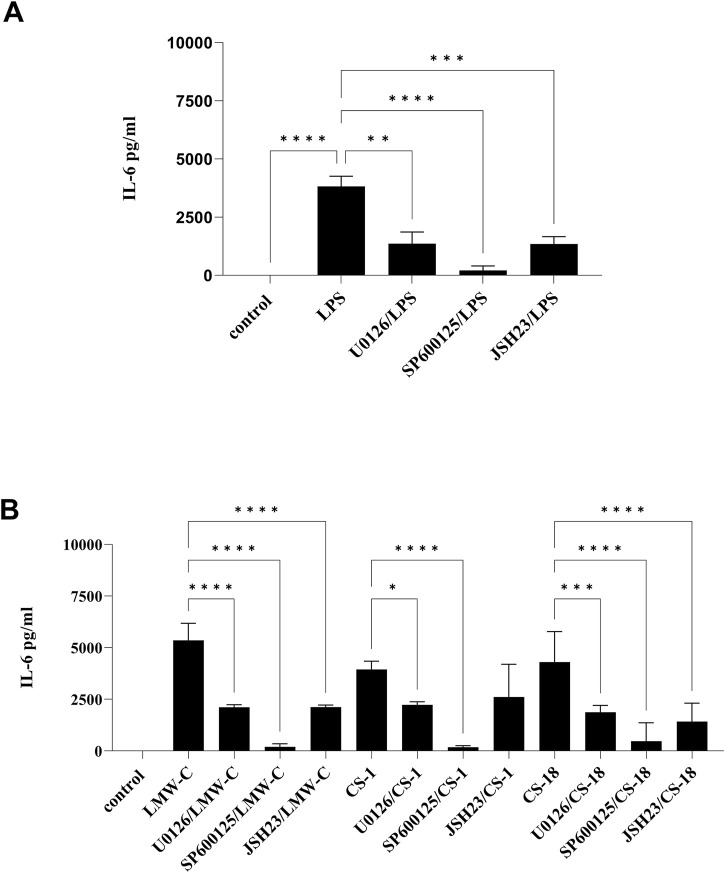
Impact of MAPK/NF-κB/iNOS inhibitors on IL-6 production in BMDM macrophages. To check the specificity of IL-6 production, BMDM cells (1 × 10^6^/mL) were first pretreated for 1 h with selective iNOS inhibitor S-MIU (10 µM), ERK 1/2 inhibitor U0126 (20 µM), JNK inhibitor SP600125 (10 µM), or JSH23 inhibitor (10 µM) and then cultured with positive control LPS (1 μg/mL) **(A)** or LMW-C, **CS-1,** and **CS-18** (100 μg/mL) **(B)** for 24 h in 5% CO_2_/95% air. Thereafter, supernatants were collected, and the level of IL-6 was detected by ELISA. The results represent at least two independent experiments (n = 2–4), and data are presented as the mean ± SD. One-way ANOVA test was used to examine the differences between LPS or LMW-C-treated cells in the presence or absence of inhibitors (*p ≤ 0.05, **p ≤ 0.001, ***p ≤ 0, and ****p ≤ 0.0001).

The expression and production of TNF-α and IL-6 are triggered by mitogen-activated protein kinase (MAPK) pathways, particularly the ERK and JNK pathways, and by the activation of the NF-κB factor, which is similar to the process of NO production (Arthur and Ley, 2013; Lawrence, 2009). Using selective inhibitors of the MAPK–NF-κB signaling pathways in our study confirmed the involvement of ERK1/2 and JNK kinases in regulating TNF-α (Figure 9B) and IL-6 (Figure 10B) expression in BMDM cells stimulated with LMW-C, and derivatives **CS-1, 18**, which is comparable to control activator LPS (Figures 9A, 10A). We also demonstrated the significant role of the transcription factor NF-κB in the signaling pathway activated by LMW-C and the **CS-18** derivative. Interestingly, the **CS-1** derivative showed a substantial decrease in IL-6 and TNF-α production when combined with NF-κB inhibitor JSH-23 (Figures 9B, 10B). However, this reduction was not statistically significant. This suggests the potential involvement of additional transcription factors, such as AP-1, or alternative signaling pathways that compensate for NF-κB inhibition.

**FIGURE 11:**
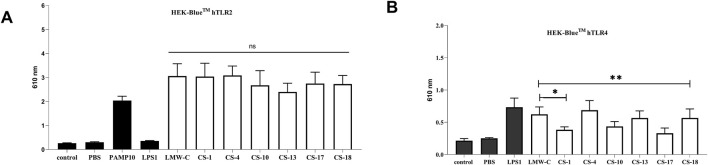
Recognition of native LMW-C and **CS-1, 4, 10, 13, 17,** and **18** derivatives by innate immune receptors. HEK Blue™ cells were seeded on the plate and stimulated with samples (10 μg/mL). The positive control for TLR2 recognition was PAMP **(A)**, whereas LPS was used as the positive control for TLR4 cells **(B)**. The data represent three independent experiments. One-way ANOVA with Dunnett’s multiple comparison test was used to compare treated cells with non-treated control (medium) and the **CS-1, 4, 10, 13, 17,** and **18** derivatives with LMW-C. *p ≤ 0.05, **p ≤ 0.01, and ns–not significant.

To investigate the role of sulfation in derivatives **CS-1, 4, 10, 13, 17,** and **18** on the activity of TLR receptors, we examined HEK-Blue™ cells stably transfected with hTLR2 or hTLR4. All the tested samples activated TLR2, and we did not observe any significant difference among sulfated derivatives when compared to LMW-C (Figure 11A). However, we observed that activation of the TLR4 receptor varied among curdlan sulfate products, as two of them (**CS-1** and **CS-17)** did not induce TLR4 activation (Figure 11B). Interestingly, this effect could not be simply ascribed to the amount of sulfate groups on the polysaccharide backbone as **CS-1** and **CS-17** showed neither the lowest nor the highest total DS values among the tested derivatives (Tables 1, 2; Figure 4). Subtler structural features could be involved; however, they cannot be unveiled at the current state of the art.

The induction of IL-6, TNF-α, and NO indicates the activation of classical inflammatory pathways and suggests the involvement of TLRs (Aizawa et al., 2018; Liu et al., 2019). Therefore, it is plausible that LMW-curdlan and its derivatives **CS-1** and **CS-18** activate TLRs, which is consistent with literature reports describing polysaccharides as ligands for pattern recognition receptors (PRRs) (Kanjan et al., 2017; Li et al., 2022; Stothers et al., 2021). However, the absence of an immunostimulatory effect observed with the other LMW-C derivatives may suggest an altered affinity for TLR2 or TLR4 receptors. This could be because of chemical modifications that change the molecule’s conformation, thereby preventing receptor activation. When analyzing interactions with LPS, a potent TLR4 agonist, the co-stimulation test can be used to assess the effect of LMW-C and its derivatives on the activation of TLR4-mediated inflammatory pathways. The lack of inhibitory activity observed when LPS and the tested compounds were administered simultaneously may result from receptor competition or the short duration of action of these substances.

During the initial 5-h incubation period, LMW-C and its derivatives significantly reduced the NO levels in a dose-dependent manner. In contrast, unmodified LMW-C significantly increased the IL-6 levels. This may indicate an early form of cell “conditioning,” possibly through the modulation of TLR2 or TLR4 expression. Overall, these results suggest that the immunological activity of macrophages is significantly impacted by chemical modifications to LMW-C, and that its derivatives may exert immunomodulatory effects, particularly following prior exposure. This effect could help in preventing excessive TLR4 activation by LPS, highlighting the potential of these substances as candidates for modulating the inflammatory response.

However, this study also has certain limitations. First, there is a need to conduct direct investigations of TLR2 and TLR4 activation, such as studies using knockout cell lines or reporter assays. It is also important to include the analysis of the dectin-1 receptor, as it can induce inflammatory responses in macrophages both independently and in cooperation with TLR2 and/or 4. The presented analysis of signaling pathways regulating NO and cytokine production, based on the use of selective inhibitors of MAP kinases, NF-κB, and iNOS, requires further validation through gene expression studies or assessment of protein phosphorylation levels using techniques such as Western blotting. This work is planned for the near future and will be published elsewhere.

## 4 Conclusion

A comprehensive study of the regioselective sulfation of LMW-C was accomplished in this work by means of three complementary semi-synthetic strategies based on (i) direct, mild sulfation reactions of only the most reactive hydroxyls, (ii) desulfation of the most reactive sulfate groups of a persulfated LMW-C derivative, or (iii) multistep methods relying on the regioselective installation of trityl ether or benzoyl ester protecting groups at the less hindered sites, followed by sulfation and deprotection. The three approaches produced a set of 12 curdlan sulfate derivatives, which were characterized in terms of total DS and sulfation pattern (DS-2, DS-4, and DS-6) by means of ^1^H- and 2D-NMR techniques. They complemented the six semi-synthetic derivatives that were already reported by us (Vessella et al., 2021a) to give a significantly broad coverage of the theoretically possible structural features. Indeed, among the semi-synthesized derivatives, the total DS value spanned from 0.34 to 3.00, whereas DS-2, DS-4, and DS-6 all ranged from 0 to 1. Six derivatives were selected from the set as representatives of such broadly variable structural features and then subjected to a preliminary screening of their immunomodulatory effects on pro-inflammatory TNF-α, pleiotropic IL-6, anti-inflammatory IL-10, and NO production by BMDM cells in comparison with the starting LMW-C polysaccharide. Some differences were detected not only between LMW-C and its sulfated derivatives but also among the set of tested curdlan sulfate products. This could suggest a role of the sulfation degree and sulfation pattern in the immunomodulant activity, which will be investigated further in the near future by additional immunological studies and molecular docking investigation of the TLR2 and TLR4 binding mode of the sulfated LMW-C derivatives.

## Data Availability

The original contributions presented in the study are included in the article/Supplementary Material; further inquiries can be directed to the corresponding author.
