# Supplementary material for: Three semi-synthetic approaches to a set of curdlan sulfate polysaccharides with different sulfation patterns
Source: Front Mol Biosci. 2025 Sep 10;12:1635564. doi: 10.3389/fmolb.2025.1635564 (PMC12456920; doi:10.3389/fmolb.2025.1635564)
Supplement: Supplementary file 1 [file Supplementaryfile1.docx]

**Three semi-synthetic approaches to a set of curdlan sulfate polysaccharides with different sulfation patterns**

**Supporting Information**

Fabiana Esposito,^1^ Agnieszka Zabłocka,^2^ Serena Traboni,^1^ Alfonso Iadonisi,^1^ Sabina Górska,^2^ Emiliano Bedini ^1^

*^1^Department of Chemical Sciences, University of Naples Federico II,*

*Complesso Universitario Monte S.Angelo, via Cintia 4, I-80126 Napoli, Italy*

*^2^L. Hirszfeld Institute of Immunology and Experimental Therapy, Polish Academy of Sciences,*

*Weigla 12, 53-114 Wrocław, Poland*

**Table of Contents**

**Figure S1**: ^1^H- and 1D-DOSY NMR spectra of **1**  S-3

**Figure S2**: ^1^H- and ^1^H,^13^C-DEPT-HSQC NMR spectra of **CS-5**  S-3

**Figure S3**: ^1^H- and COSY NMR spectra of **CS-5**  S-4

**Figure S4**: ^1^H- and COSY NMR spectra of **CS-9**  S-4

**Figure S5**: ^1^H- and ^1^H,^13^C-DEPT-HSQC NMR spectra of **CS-10**  S-5

**Figure S6**: ^1^H- and COSY NMR spectra of **CS-10**  S-5

**Figure S7**: ^1^H- and ^1^H,^13^C-DEPT-HSQC NMR spectra of **CS-11**  S-6

**Figure S8**: ^1^H- and ^1^H,^13^C-DEPT-HSQC NMR spectra of **CS-12** S-6

**
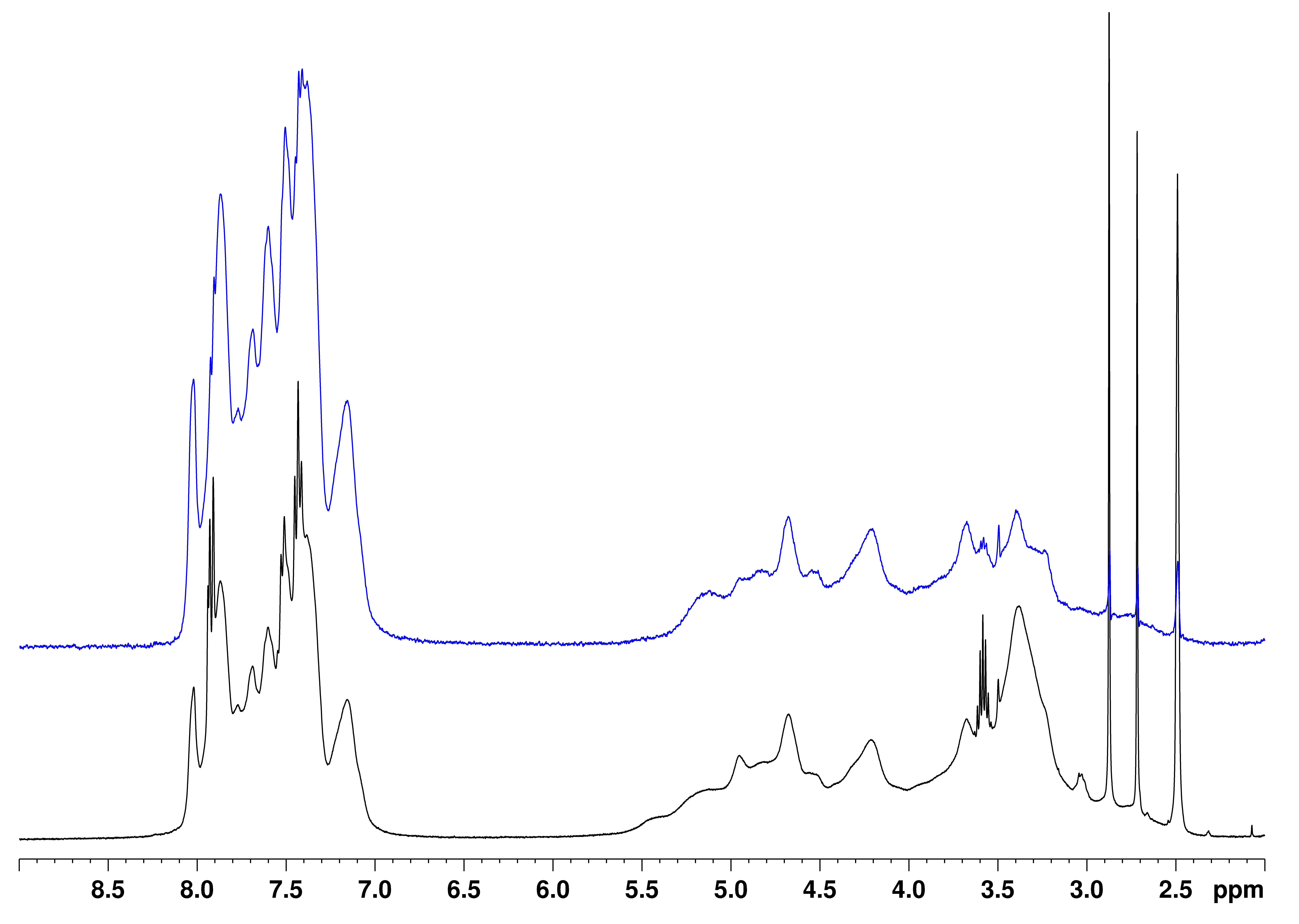
**

Figure S1: ^1^H-NMR (in black) and 1D-DOSY NMR (in blue) spectra (400 MHz, 298K, DMSO-*d_6_*) of **1**


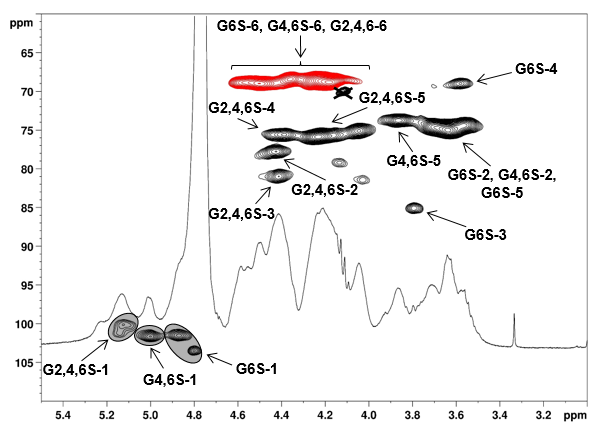


Figure S2: ^1^H- and ^1^H,^13^C-DEPT-HSQC NMR spectra (400 MHz, 298K, D_2_O) of **CS-5** with assignment of the main signals (G6S = 6-O-sulfated-Glc, G4,6S = 4,6-di-O-sulfated-Glc, G2,4,6S = 2,4,6-tri-O-sulfated-Glc; DEPT-HSQC signals enclosed in grey circles were integrated for DS-2 and DS-4 measurement)


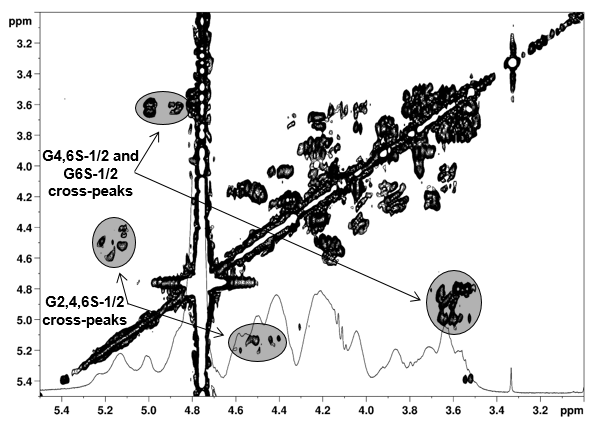


Figure S3: ^1^H- and COSY NMR spectra (400 MHz, 298K, D_2_O) of **CS-5** with assignment of the H-1/H-2 cross-peaks (G6S = 6-O-sulfated-Glc, G4,6S = 4,6-di-O-sulfated-Glc, G2,4,6S = 2,4,6-tri-O-sulfated-Glc)


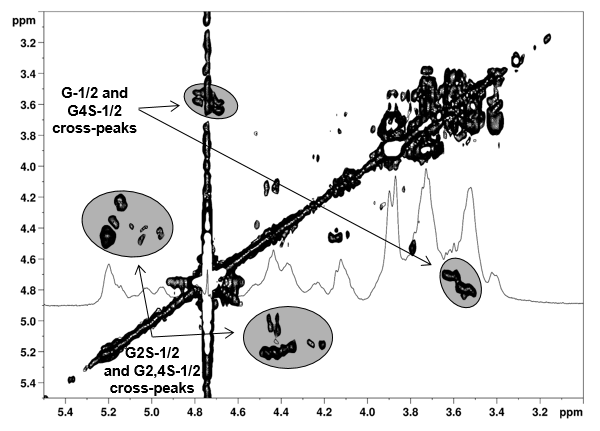


Figure S4: ^1^H- and COSY NMR spectra (400 MHz, 298K, D_2_O) of **CS-9** with assignment of the H-1/H-2 cross-peaks (G2S = 2-O-sulfated-Glc, G4S = 4-O-sulfated-Glc, G2,4S = 2,4-di-O-sulfated-Glc)


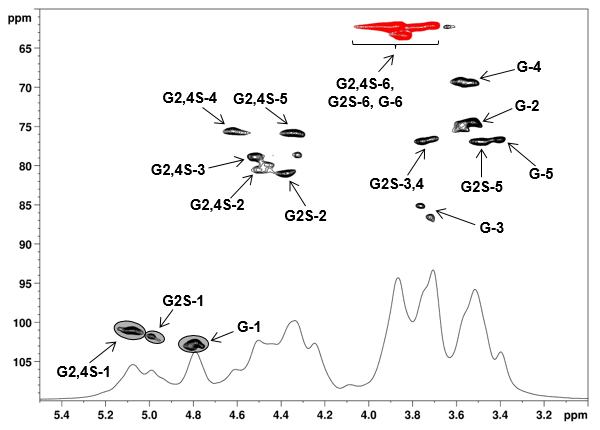


Figure S5: ^1^H- and ^1^H,^13^C-DEPT-HSQC NMR spectra (600 MHz, 298K, D_2_O) of **CS-10** with assignment of the main signals (G = Glc, G2S = 2-O-sulfated-Glc, G2,4S = 2,4-di-O-sulfated-Glc; DEPT-HSQC signals enclosed in grey circles were integrated for DS-2 and DS-4 measurement)


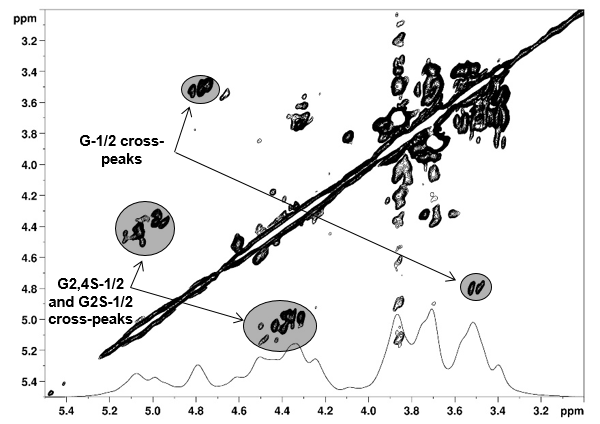


Figure S6: ^1^H- and COSY NMR spectra (600 MHz, 298K, D_2_O) of **CS-10** with assignment of the H-1/H-2 cross-peaks (G = Glc, G2S = 2-O-sulfated-Glc, G2,4S = 2,4-di-O-sulfated-Glc)


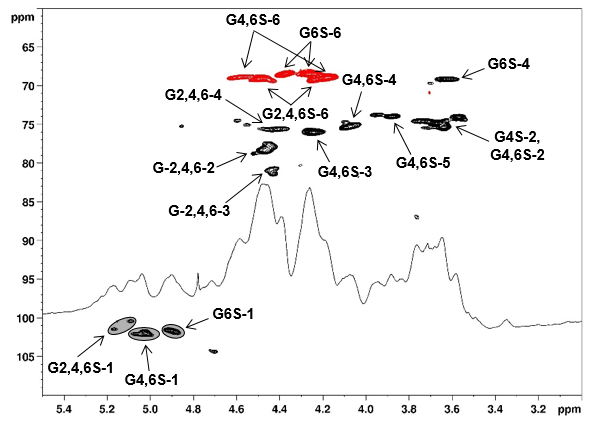


Figure S7: ^1^H- and ^1^H,^13^C-DEPT-HSQC NMR spectra (600 MHz, 298K, D_2_O) of **CS-11** with assignment of the main signals (G6S = 6-O-sulfated-Glc, G4,6S = 4,6-di-O-sulfated-Glc; G2,4,6S = 2,4,6-tri-O-sulfated-Glc; DEPT-HSQC signals enclosed in grey circles were integrated for DS-2 and DS-4 measurement)


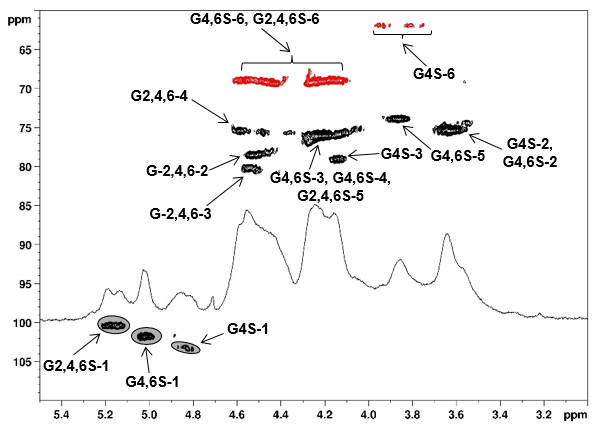


Figure S8: ^1^H- and ^1^H,^13^C-DEPT-HSQC NMR spectra (400 MHz, 298K, D_2_O) of **CS-12** with assignment of the main signals (G4S = 4-O-sulfated-Glc, G4,6S = 4,6-di-O-sulfated-Glc, G2,4,6S = 2,4,6-tri-O-sulfated-Glc; DEPT-HSQC signals enclosed in grey circles were integrated for DS-2 and DS-6 measurement)
